# Genome Trees from Conservation Profiles

**DOI:** 10.1371/journal.pcbi.0010075

**Published:** 2005-12-16

**Authors:** Fredj Tekaia, Edouard Yeramian

**Affiliations:** 1 Unité de Génétique Moléculaire des Levures (URA 2171 CNRS and UFR927 Univ. P.M. Curie), Institut Pasteur, Paris, France; 2 Unité de Bio-Informatique Structurale (URA 2185 CNRS), Institut Pasteur, Paris, France; University of California San Diego, United States of America

## Abstract

The concept of the genome tree depends on the potential evolutionary significance in the clustering of species according to similarities in the gene content of their genomes. In this respect, genome trees have often been identified with species trees. With the rapid expansion of genome sequence data it becomes of increasing importance to develop accurate methods for grasping global trends for the phylogenetic signals that mutually link the various genomes. We therefore derive here the methodological concept of genome trees based on protein conservation profiles in multiple species. The basic idea in this derivation is that the multi-component “presence-absence” protein conservation profiles permit tracking of common evolutionary histories of genes across multiple genomes. We show that a significant reduction in informational redundancy is achieved by considering only the subset of distinct conservation profiles. Beyond these basic ideas, we point out various pitfalls and limitations associated with the data handling, paving the way for further improvements. As an illustration for the methods, we analyze a genome tree based on the above principles, along with a series of other trees derived from the same data and based on pair-wise comparisons (ancestral duplication-conservation and shared orthologs). In all trees we observe a sharp discrimination between the three primary domains of life: Bacteria, Archaea, and Eukarya. The new genome tree, based on conservation profiles, displays a significant correspondence with classically recognized taxonomical groupings, along with a series of departures from such conventional clusterings.

## Synopsis

Since Darwin's *Origin of Species* and Haeckel's *Tree of Life,* systematic biology has attempted to classify species into “family trees.” Genomics has provided a new framework permitting descriptions of sibling relations between species on the basis of their complete genetic blueprints. While trees based on single genes (rRNA), or limited numbers of genes have been useful, genome trees derived from complete genome comparisons should lead to more complete pictures of phylogenetic relations between various organisms. In order to reach such a global vision, procedures to establish sibling relationships should depend on an overall comparison that captures the evolutionary fates of proteins jointly in multiple genomes. This paper aims to establish a methodological basis to use genuine multidimensional procedures in the construction of genome trees. This approach completes the derivation of trees based on more classical techniques of pair-wise comparison between species. The authors survey classification schemes emerging from this approach, which either supports traditional views, such as the separation between the three phylogenetic domains Bacteria, Archaea, and Eukarya, or challenges them by suggesting, for example, intermingled clusterings of Proteobacteria with various other bacterial species.

## Introduction

Genomes contain many levels of phylogenetic information. As well as sequences of nucleotides and amino acids, complete genomes also contain structural information on the order of genes [1], nucleotide usage patterns [2], and amino-acid composition [3,4]. The evolution of genome content has become a central issue in comparative genomics revealing major evolutionary events including gene loss, gene acquisition through horizontal transfer [5–10], transfer of mitochondrial DNA sequences to the nucleus [11], and gene duplication [12–14]. Such events tend to undermine the concept of “the universal phylogenetic tree” since no single gene tree can reflect evolution in all species. Moreover, since single gene families represent only a minor fraction of genomic information, it has been argued that focusing on single genetic elements (such as rRNA genes) can be inadequate for an integrative analysis of complete character complexes and the construction of phylogenetic trees of whole organisms. Accordingly, various integrative procedures have been designed to overcome these difficulties [15–17]. For example, the construction of “phylogenomic trees” involves the use of longer and richer datasets, obtained by joining large sequence stretches or concatenated proteins common to several species [18,19]. In another direction, the construction of “supertrees” relies on several individual gene trees [20,21].

Genome trees integrate information of potential evolutionary significance, based on comparisons of gene similarities, from whole genome content. Thus, the various proposed genome trees reflect global similarities based on the presence or absence of genes, gene families, protein folds, amino acid patterns [22–28], or gene order [29,30]. More recently, genome trees have been based on protein domain contents [31] or “genome conservation” [32]. The rationale in making phylogenetic inferences from such information is that shared similarities in the organization of two genomes should correspond to inherited features from a common ancestor. The methods used to assess information from complete genomes rely on the occurrence of shared orthologs or shared gene families, as measures of similarity. However, despite their major advantages over single-gene trees, the derivation of genome trees still suffers from a series of limitations and difficulties, essentially relevant to the choice of the data, and to the adequacy of the methods used to analyze them.

The primary information used to construct genome trees reflects phylogenetic relations and evolutionary events relevant to gene transfer, gene loss, and acquisition. It has necessarily mixed origins. The construction of robust genome trees still remains in many ways an unachieved goal. The problems and limitations encountered in the construction of genome trees are of different origins. For the genome data, biodiversity is not homogeneously represented in the various branches of the three domains of life. The assessment and estimation of gene acquisition via duplication, horizontal transfer, or other processes [8,33,34] remains difficult despite recent reappraisals [13,35–39] and new methods adopted to better treat them (derivation of genomic trees [40,41], or genomic non-tree topologies [42,43]). Finally, tree building methodologies have so far not fully exploited the multidimensional nature of the evolutionary genomic information, obtained jointly across several species.

In the context of these limitations, we introduce here methods to derive genome trees based on “conservation profiles,” taking fully into account the high dimensional nature of the data and the multidimensional nature of the evolutionary histories of proteins. Indeed, the conservation profile of a given protein captures an evolutionary history, expressed as an *n-*component vector detailing the presence or absence of homologs, in each of the *n* considered species. Through the multidimensional structure of conservation profiles, the evolutionary history of proteins is thus observed jointly across *n* species: proteins with identical conservation profiles can be associated with identical evolutionary histories. From the complete set of considered proteomes, the set of distinct conservation profiles is indicative of the various evolutionary histories.

On methodological grounds, we used multivariate analysis for deriving genome trees from conservation profiles. Such derivations highlight some difficulties in the handling of conservation profiles, as representation of phylogenetic histories. These difficulties are discussed in some detail, paving the way for possible improvements. On the one hand, resorting to conservation profiles permits reduction of informational redundancy by retaining only distinct conservation profiles. On the other hand, analysis of conservation profiles from the proteomes of 99 complete genomes showed that many proteins (in the same or in different species) share identical evolutionary histories, leading to a very small set of shared distinct profiles (associated with at least two proteins from two distinct species). The criteria for the derivation of trees from profiles are thus not trivial, with various possible compromises on stringency. Stated otherwise, should we consider the full set of all distinct conservation profiles or retain only the core set of shared distinct conservation profiles? We explored these possibilities by constructing a genome tree based on the core set of shared distinct profiles. One step further, to reasonably relax the strict restriction to shared distinct profiles, we considered the whole set of distinct conservation profiles, resorting to Jaccard similarity scores between pairs of species (as calculated from the whole set of distinct conservation profiles), and also derived the corresponding tree.

Beyond the methodological derivations, for a first exploration of this new type of genome tree, we analyze in some detail the topology of the tree based on profiles obtained from Jaccard scores. This analysis is performed in parallel with the analysis of other trees obtained from the same set of genomic data: (a) a genome tree based on ancestral duplication and ancestral conservation weights (an updated version of the genome tree presented in [23]) and (b) a genome tree based on shared orthologs. This comparative analysis reveals very stable features and clusters, along with a series of variations following the trees. All trees discriminated clearly between the three phylogenetic domains of life. A series of variable features, amongst the trees, appears to reflect rather faithfully various alternative hypotheses associated with debated phylogenetic clusterings. This observation is interpreted in part in the light of possible interplays between phylogeny and genome dynamics.

## Results

**Figure 1:**
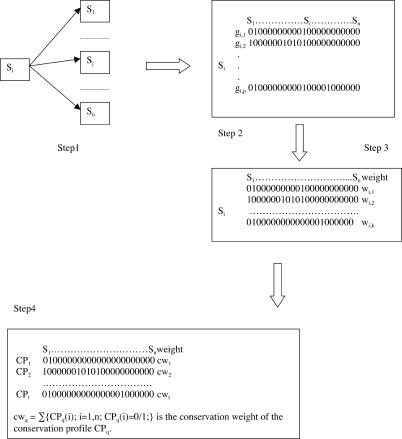
Determination of Distinct Conservation Profiles for Proteins The flow chart details steps in the determination of distinct conservation profiles for proteins in 99 predicted proteomes. The steps are as follows: (A) Step 1: Species-specific predicted proteome comparisons. Each protein sequence of species S_i_ (see list in Table S1) was compared to each database of all proteins from each surveyed species, using the BLASTP program (See Materials and Methods). Best significant matches in each of the considered species were determined. The original 541,880 protein sequences, lead to 442,460 non-specific proteins (i.e., 81.7%). Fractions of ancestral duplication and ancestral conservation were determined. Each protein was then described by a vector whose components are zeros (no matches) or best significant matches whenever hits occur in each of the considered species. From the list of proteins and their corresponding best hits, pairs of orthologs were determined by looking for reciprocal best significant hits. (B) Step 2: Protein conservation profiles. In each species S_i_, the conservation profile of each protein k, denoted g_i,k_, is represented by a *n-*component vector of ones and zeros describing its pattern of conservation across all species. Each vector associated with a conservation profile is of size 99, corresponding to the total number of surveyed species (in the order indicated in Table S1). (C) Step 3: Distinct conservation profiles. In each species S_i_, identical conservation profiles were represented by a single representative, leading to the set of distinct conservation profiles. In this simplification, a “weight” is associated to a given conservation profile, as the total number of proteins with that profile. For example 3,154 distinct conservation profiles were found in *S. cerevisiae,* 5,690 in *A. gambiae,* 6,225 in *H. sapiens,* and 1,716 in *P. falciparum.* (D) Step 4: Overall characterization of distinct conservation profiles. The overall set of distinct conservation profiles amounted to 184,130 profiles. The “conservation weight” of each conservation profile is determined, as the total sum of 1.

The large-scale predicted proteome comparisons (see Materials and Methods) permit determination of conservation profiles for each protein of *n* considered species (*n* = 99; Table S1 and Figure 1, steps 1 and 2). For each protein, the conservation profile is represented by an *n-*component vector of zeros and ones, which describes its conservation pattern across the *n* species (zero corresponds to the absence and one to the presence of a homolog in the various species). The conservation profile of a protein sequence can be associated with its evolutionary history in a multidimensional genome space. This mathematical definition of “conservation profile” is identical to that of “phylogenetic profile” [44–45] as it is based on the same vector. The terms “phyletic pattern” and “phylogenetic pattern” have also been used to describe the same vector [46]. Here, we prefer “conservation profile” since it refers explicitly to the comparison process. The “evolutionary profile” underlying such multidimensional comparisons, can indeed be associated with evolutionary processes (such as horizontal transfer or duplication) rather than purely vertical inheritance (i.e., phylogeny).

### Distinct Conservation Profiles

The large-scale proteome comparisons for the 99 completely sequenced genomes analyzed involved a total of 541,880 proteins (Table S1). The comparisons led to 442,460 non-specific proteins with non-trivial conservation profiles (i.e., with at least one homolog outside their own proteome), resulting in 184,130 distinct conservation profiles, which retained only one representative from each set of identical conservation profiles (Figure 1, steps 3 and 4). Thus, distinct conservation profiles represent 41.6% of the total set of non-specific proteins. One step further, we consider the core subset of shared distinct conservation profiles, associated with at least two proteins from distinct species. This core subset reduces to 24,044 profiles, which represent only 5.4% of the whole set of non-specific conservation profiles and 13% of the set of distinct conservation profiles.

These data provide several possible choices for the derivation of trees from conservation profiles. Based on maximal redundancy reduction, we can adopt the core reduced subset of shared distinct conservation profiles. Alternatively, this choice could be seen as too reductive, since it discards information contained in the 160,086 distinct conservation profiles associated with only one species, which correspond to one or several proteins from that species. It is then possible to derive trees that consider the additional information in this set of profiles, with potential relevance to ancestry signals.

**Figure 2:**
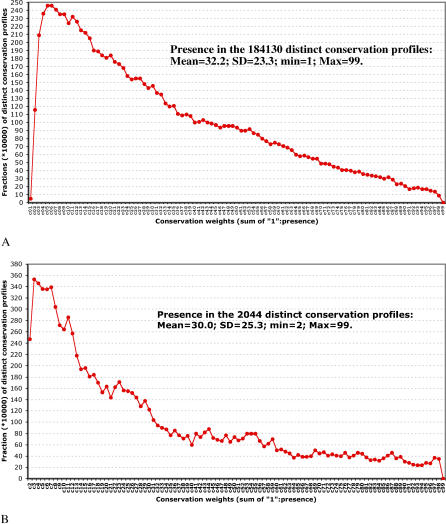
Distinct Conservation Profiles and Corresponding Weights (A) Distribution of the whole set of distinct conservation profiles (184,130) according to the 99 possible weight classes varying from one to 99. (B) Similar distribution restricted to the subset of distinct conservation profiles (2,044) associated with proteins from at least two species.

In the light of these different choices, it may be of interest to quantify the characteristics of information contained in the distribution of profiles. Thus, each of the 24,044 shared distinct profiles, associated with at least two species, involved an average of 11.9 proteins. The classification of profiles according to relative “conservation weights” (or the total number of occurrences of 1 in the given profile; this number could vary between 1 and 99), led to an average weight of 30 (SD = 25.3). For most conservation profiles, conservation weights ranged between three and seven. Overall, the distribution of the number of profiles decreased uniformly as conservation weights increased (Figure 2A and 2B). Finally, for the set of 184,130 distinct conservation profiles, similarities between pairs of species were evaluated from the Jaccard score (see formula in Materials and Methods).

### Genome Trees: Similarity Matrices

**Figure 3:**
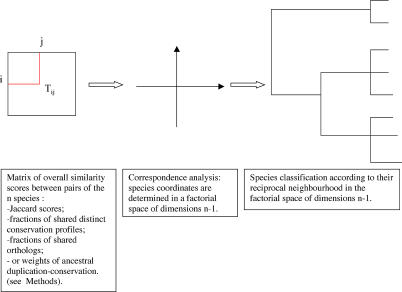
Genome Tree Construction The flow chart details the three steps in genome tree construction. In the first step a data matrix is constructed, based on overall similarity scores between pairs of species (i.e., the fraction of shared distinct conservation profiles, Jaccard scores, fraction of shared homologs, or ancestral duplication conservation weights). In the second step, correspondence analysis is performed on the data tables, for constructing the corresponding factorial spaces (orthogonal systems of dimensions *n*–1, with *n* the number of lines in the considered matrices). In the third step the genome trees are derived based on the reciprocal neighboring of the species from their Euclidean distances, as calculated in the factorial spaces.

The various genome trees considered here were derived using a common rationale, as shown in Figure 3. First, a data matrix T was constructed from similarity scores measuring the relatedness of each pair of species (see Materials and Methods): fractions of shared distinct conservation profiles, Jaccard scores, fractions of shared orthologs, and finally, ancestral duplication-conservation weights. Secondly, correspondence analysis was performed [47,48] to construct an orthogonal system, and to represent the *n* species in the corresponding factorial space of dimensions *n*–1. Finally, each resulting genome tree was derived, based on the reciprocal neighboring of the species, using Euclidean distances calculated from coordinates in the factorial space. We will consider in some detail the genome tree associated with Jaccard scores (that we term *profiles tree*), comparing it with the three other trees (*minimal profiles tree,* Figure S1, based on shared distinct conservation profiles; *orthologs tree,* Figure S2, based on shared orthologs; and *conservation tree,* Figure S3, based on ancestral duplication and conservation weights). We highlight features that seem to be stable in these various trees and those that are most variable.

We note that all trees are derived from the same set of genomic data, and depend on multidimensional or pair-wise conservations, thus reflecting potentially different evolutionary relationships. More precisely, conservation profiles reflect detected evolutionary relationships across all surveyed species (multidimensional evolutionary signatures), whereas the orthologs and the ancestral duplications-conservations reflect detected evolutionary relationships between pairs of species. Also, following the terminology in [27], the *conservation tree* is relevant to the “homolog method” and the *orthologs tree* is relevant to the “ortholog method.” For the new *profiles tree* we could similarly refer to the “conservation profiles method.”

### Tree Topologies and Clusterings of Species

**Figure 4:**
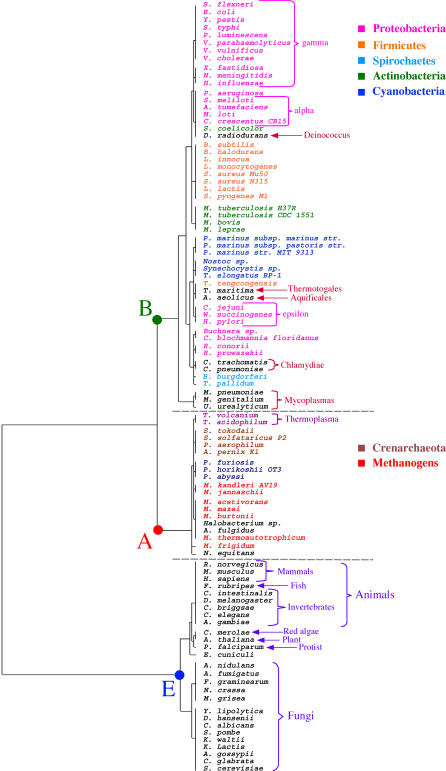
Genome Tree *Profiles tree* based on Jaccard scores as obtained from the whole set of distinct conservation profiles.

**Figure 5:**
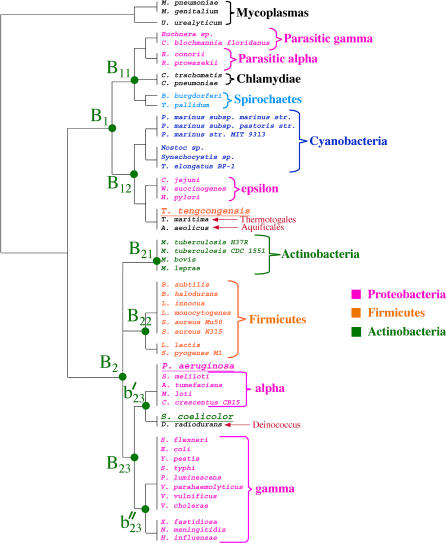
Bacterial Branch *Bacteria subtree* (see Materials and Methods), based on the restriction of the Jaccard scores matrix to the lines corresponding to bacterial species.

**Figure 6:**
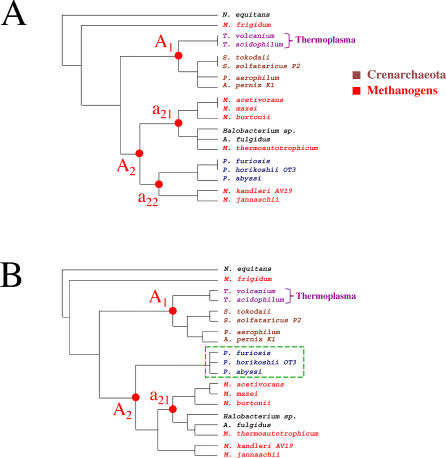
Archaeal Branch The archaeal branch is represented in more detail with (A) *archaea subtree* (see Materials and Methods), based on the restriction of the Jaccard scores matrix to the lines corresponding to archaeal species, and (B) *archaea only subtree* based on the restriction of the Jaccard scores matrix to the lines and the columns corresponding to archaeal species.

**Figure 7:**
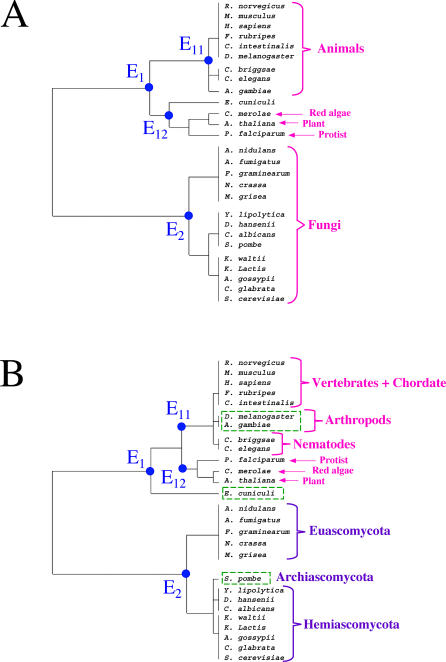
Eukaryal Branch The eukaryal branch is represented in more detail with (A) *eukarya subtree* (see Materials and Methods) based on the restriction of the Jaccard scores matrix to the lines corresponding to eukaryal species, and (B) *eukarya only subtree* based on the restriction of the Jaccard scores matrix to the lines and the columns corresponding to eukaryal species.

The first striking observation is that the three domains of life are clearly separated in the *profiles tree* (Figure 4), with the branching of Archaea with Bacteria. This separation, as well as the Archaea-Bacteria branching, apparently corresponds to very stable features throughout the different trees (Figures S1, S2, and S3). At such a global level, the only difference between the various trees concerns variable levels of resolution. With this respect, as illustrated here for the *profiles tree* (Figure 4), enhanced resolutions can be achieved by considering partial trees, which can be associated, for example, with each one of the three domains of life, separately. In what follows, we consider two different types of such partial trees (see Materials and Methods for more details), in which we restrict the construction of the partial trees to the species of a given domain of life. Still taking into account the comparisons between all species in the three domains, the restrictions are only at the level of similarity matrices. Thus, from the similarity matrix of the *profiles tree,* by restricting ourselves to the lines associated with the species in the respective domains we derive the *bacteria subtree, archaea subtree,* and *eukarya subtree* (Figures 5, 6A, and 7A, respectively). By further restricting the matrix at the level of the columns as well (with lines and columns corresponding to species in a given domain), we define the *archaea only subtree* and *eukarya only subtree* (Figures 6B and 7B).

### Bacterial Branch

#### General structure.

Clusters in the bacterial branch follow accepted taxonomical groupings rather closely, with only a few departures. The Mycoplasmas are the most distant cluster (as further illustrated in the *bacteria subtree,* Figure 5). Beyond the out-branched Mycoplasmas, the bacterial branch splits into two nodes (B_1_ and B_2_, on the *bacteria subtree,* Figure 5). Following this major organization, some classically accepted taxonomical groups are homogeneously clustered, whereas others —such as the Proteobacteria— are scattered throughout several nodes and branches. We next consider in more detail the organization of the bacterial branch according to classical taxonomical classifications. Due to various intermingled clusters the analysis follows the hierarchical structure of the tree, rather than strict taxonomical classifications.

The B_1_ node (Figure 5) is bifurcated, with two clearly separated branches at nodes B_11_ and B_12_. The B_11_ node clusters together three (homogeneous) subclusters: (a) the parasitic alpha and gamma Proteobacteria, (b) the Chlamydiae, and (c) the Spirochaetes. The B_12_ node clusters two clearly separated subgroups: (a) the Cyanobacteria and (b) a clustering of the epsilon species with a composite group comprising *Thermoanaerobacter tengcongensis* (underlined as separated from the other Firmicutes), *Thermotoga maritima* and a *Aquifex aeolicus.*

The B_2_ node (Figure 5) splits into three branches, at the nodes B_21_, B_22_, and B_23_. The B_21_ node joins together all Actinobacteria (with the exception of *Streptomyces coelicolor,* underlined in the B_23_ node). The B_22_ node unites all the Firmicutes (with the exception of *T. tengcongensis,* as already mentioned). The B_23_ node splits into two subclusters: (a) the node b'_23_ groups alpha Proteobacteria (with the underlined gamma species *Pseudomonas aeruginosa*) along with the association of *S. coelicolor* (Actinobacteria) and *Deinococcus radiodurans* (Deinococcus) and (b) the b''_23_ node unites gamma Proteobacteria. In this overall organization we note that the b'_23_ node joins a series of soil/plant associated bacteria, from different phylogenetic groups but with common lifestyle features. This clustering unites the free-living *S. coelicolor* (Actinobacteria; which has developed a large coding potential involving many proteins implicated in regulatory functions), with the pathogenic *P. aeruginosa* (with free-living capacities), and a series of rhibozomal microsymbionts (alpha Proteobacteria). This clustering overlaps rather sharply with those observed, for example, on the basis of transport capabilities [49], since the concerned organisms “have more ABC transporters than any other sequenced organisms” [49]. We also note (as in [49]) that such clustering is uncorrelated with genome size. The genome of *D. radiodurans* is about 3.3 Mega bases while that of *S. coelicolor* is about 6.2 Mega bases, for example.

#### Stabilities versus variabilities in the background of alternative phylogenetic hypotheses.

The out-grouping of the Mycoplasmas does not seem to be a stable feature across the trees we consider. In the *minimal profiles tree* as well as in the *orthologs tree* the most distant cluster concerns Actinobacteria (Figures S1 and S2). Also, at this level, the analyses are not consistent with other work, which suggests that either the Thermotogales or the Aquificales are the most out-grouped of the bacterial branch [18,26].

The scattering of the Proteobacteria at various nodes of the bacterial branch is found in all the trees considered here (see also Figures S1, S2, and S3). This feature is consistent with conclusions in many analyses [26,50], and contradicts monophyletic proteobacterial clusters observed in certain studies [18,31]. At a more detailed level, several associations between various Proteobacteria seem to be very stable, such as the association (node b'_23_, Figure 5) of the pathogenic *P. aeruginosa* (gamma species) with a series of rhibozomal microsymbionts (alpha species). This cluster seems to be systematically clustered with the free-living Actinobacteria *S. coelicolor* in all trees examined here. On the other hand, the association of *D. radiodurans* with this cluster varies according to the chosen tree. In the *minimal profiles tree* (Figure S1) and in the *conservation tree* (Figure S3), the Actinobacteria *Mycobacterium leprae* joins *S. coelicolor,* and surprisingly unites a highly decaying species with a series of species with extended repertoires for adaptation.

Other composite associations also seem to be very stable, such as that concerning the Spirochaetes, the Chlamydiae, and the parasitic Proteobacteria (node B_11_ in Figure 5). Interestingly, this association is observed not only in the various trees here, but also in other analyses [26].

Concerning the Firmicutes, we note that *T. tengcongensis* is separated from the other Firmicutes in all trees. This separation may reflect the ambiguous status of this species in traditional classifications. While empirical definitions suggest that it is gram-negative, analysis of the complete genome revealed that *T. tengcongensis* “shares many genes characteristic of gram-positive bacteria” [51]. Similar observations have been reported in trees in recent studies [50].

### Archaeal Branch

#### General structure.

In the archaeal branch, the hyperthermophilic *Nanoarchaeum equitans* and the psychrophilic *Methanogenium frigidum* are out-grouped. We note that *N. equitans* has been assigned recently to a novel archaeal phylum (“Nanoarchaeota” [52]).

Beyond these out-grouped species, the archaeal branch displays little resolution in the *profiles tree* (Figure 4), but is bifurcated with the enhanced resolution of the *archaea subtree* and *archaea only subtree* (nodes A1 and A2, in Figure 6A and 6B). This bifurcated structure does not follow the Crenarchaeota/Euryarchaeota separation, even if the four Crenarchaeota species are clustered together.

The A1 node (Figure 6A and 6B) clusters the Crenarchaeota species together with the Thermoplasma. The organization of the A2 node varies between the *archaea subtree* (Figure 6A) and the *archaea only subtree* (Figure 6B). In the *archaea subtree,* the node A2 bifurcates with the node a_21_ clustering together a series of Methanogens with *Halobacterium* sp. and *Archaeoglobus fulgidus,* and the node a_22_ clustering together the Pyrococcus species with two Methanogen species *(Methanopyrus kandleri* and *Methanopyrus janaschii).* In the *archaea only subtree,* the Pyrococcus cluster shifts with respect to the *archaea subtree,* becoming out-branched from a mainly Methanogens cluster, joining the a_21_ node of the *archaea subtree* with the remaining two Methanogens *(M. kandleri* and *M. janaschii).*

#### Stabilities versus variabilities in the background of alternative phylogenetic hypotheses.

In terms of major clades, these analyses do not support the classification of the Archaea after the Crenarchaeota/Euryarchaeota separation, despite the co-clustering of the Crenarchaoeta species observed in the *profiles tree.* Clustering together of the Crenarchaoeta is not always observed in these trees (see for example the *minimal profiles tree;* Figure S1). This conclusion on Crenarchaeota/Euryarchaeota is consistent with various other analyses (such as in [26], where Crenarchaoeta cluster with the Thermoplasma). In fact, recent genome tree studies have rarely supported Crenarchaeota/Euryarchaeota separation (moderately supported in [18], on the basis of a single species, *Aeropyrum pernix*). As for the novel archaeal phylum “Nanoarchaeota,” it is difficult to draw firm conclusions here since it concerns a single species *N. equitans* (out-grouped in the *minimal profiles tree,* but not in the *orthologs tree;* see Figures S1 and S2).

A more detailed study of the branch reveals an inconsistency between the *archaea subtree* (Figure 6A) and *archaea only subtree* (Figure 6B) for the positioning of the Pyrococcus. These data could therefore either support or contradict potential monophyly of Methanogens. This doubt about the appropriate position for the Pyrococcus is confirmed by the other trees. In the *orthologs tree* (Figure S2), for example, Pyrococcus joins the other node of the archaeal branch, with Crenarchaeota and Thermoplasma species. Of these possibilities, an out-grouping of Pyrococcus from a largely homogeneous Methanogens cluster, as in the *archaea only subtree* (Figure 6B), is consistent with the representation of [26]. *A. fulgidus* clusters with the Methanogens in all the trees considered here. In contrast, *Halobacterium* sp. does not cluster with the Methanogens in the *minimal profiles tree* or in the *conservation tree* (Figures S1 and S3). In the literature, the positioning of *A. fulgidus* relative to the other *Archaea* has been controversial, shifting from a deep-branching position toward a grouping with Methanomicrobiales and extreme halophiles [53], based on rRNA genes. However, with the completion of its genome, it was revealed that in *A. fulgidus* “all the enzymes and cofactors of methanogenesis are used, but the absence of methyl-CoM reductase eliminates the possibility of methane production by conventional pathways” [54], thus reinforcing the firm clustering consistently observed here.

### Eukaryal Branch

#### General structure.

The eukaryal branch bifurcates with two clearly separated branches (Figure 4). This structure is preserved with the enhanced resolution in the *eukarya subtree* (Figure 7A) and *eukarya only subtree* (Figure 7B). In these representations of the eukaryal branch, the first node joins together the animals (Mammals, Nematodes, Arthropods, and the Chordate *Ciona intestinalis*), along with a composite cluster comprising a red algae, a plant, and a protist. The second branch unites various fungal species.

At a more detailed level, in the *profiles tree* (Figure 4), *Encephaliltozoon cuniculi* is out-grouped in the first node. In this *profiles tree* no separations are observed in the animals cluster. Better resolution, in the *eukarya subtree* and *eukarya only subtree* (Figure 7A and 7B, respectively) reveals an unstable positioning of *E.cuniculi.* In the *eukarya subtree, E. cuniculi* is distant from the red algae-plant–protist *(Plasmodium falciparum)* cluster at the E_12_ node, whereas in the *eukarya only subtree,* it is distant from all animals at the E_1_ node (as in the *profiles tree*). For the animals, in the *eukarya subtree,* a separation appears between Nematodes and the other animals (node E_11_, with *Anopheles gambiae* out-grouped), whereas in the *eukarya only subtree* (Figure 7B, node E_11_) we observe a more precise clustering following Vertebrates along with the Chordate *C. intestinalis,* the Nematodes, and the Arthropods. At the second node (E_2_), an increasing resolution appears between the *profiles tree,* the *eukarya subtree,* and the *eukarya only subtree,* respectively. A progressive resolution is apparent in the fungi branch with the separation of *Schizosaccharomyses pombe* from the other yeasts in the *eukarya only subtree.* In this tree we obtain essentially a separation of the fungi in clusters corresponding to Euascomycota, Archiascomycota *(S. pombe),* and Hemiascomycota. In this case, the genomic subtree reflects, rather faithfully, admitted phylogenies [55], either based on limited sets of orthologous proteins (Resources for Fungal Comparative Genomics: http://fungal.genome.duke.edu) or fungal mitochondrial genome projects (Global Fungal Phylogeny: http://megasun.bch.umontreal.ca/People/lang/FMGP/phylogeny.html), with the precise positioning of the out-grouped *S. pombe* indeed varying following the studies.

#### Stabilities versus variabilities in the background of alternative phylogenetic hypotheses.

The bifurcated structure of the eukaryal branch is found consistently in the various trees considered here. At the highest level, the only observed variance is that the red algae-plant–protist cluster joins with the fungi branch in the *conservation tree* (Figure S3). It is interesting to note that at present for *eukarya,* relations between plants, animals, and fungi “have not been conclusively resolved” [56]. None of the analyses here, with a bifurcated structure of the eukaryal branch, support recent phylogenetic analyses, which imply the definition of an Opisthokonta “super-taxon” that joins animals and fungi [57,58]. With additional genomes becoming available (notably plants), it will be important to see if this bifurcated structure is further confirmed. At this large organizational level, we note that none of the analyses would link the microsporidian *E. cuniculi* with the fungi, with the exception of the *conservation tree,* where the cluster with the fungi also includes plants and other protists *(P. falciparum).* This observation is inconsistent with the “general consensus” [56] on the relation of microsporidians to fungi. However, as noticed in [56], this consensus depends essentially on phylogenies of single proteins, and is still under debate.

At a more detailed level, the classical “Coelomata hypothesis,” suggests that Arthropods are closer to Vertebrates than to Nematodes, whereas the recent “Ecdysozoa hypothesis” suggests Nematodes should be clustered with Arthropods [56]. The various representations of the eukaryal branch in the *profiles tree* do not permit discrimination between these two hypotheses. Nonetheless, the *minimal profiles tree* (Figure S1) reveals a clear clustering of Nematodes with the Arthropods, while in the *orthologs tree* and in the *conservation tree* (Figures S2 and S3), the Nematodes are out-grouped, and the Arthropods are associated with the Vertebrates. Such instabilities suggest that contradictory theories reflect different interpretations of the same data. However, instabilities might instead derive from the quality of the data and notably of annotations. Plausibly such is the case for the variable positioning of *A. gambiae* (directly clustered with *Drosophila melanogaster* only in the *eukarya only subtree*, Figure 7B), and in the *orthologs tree* (Figure S2).

## Discussion

The primary concern of our work was to derive methods to construct genome trees from conservation profiles. One challenging problem in constructing genome trees is to separate—as much as possible—phylogenetic signals from other evolutionary “noise,” deriving from gene acquisitions via horizontal transfer, duplication, and gene losses. Thus, information in protein conservation profiles may represent an especially accurate marker for genome classification, since it embeds the most conserved and meaningful evolutionary signals, captured jointly in the whole set of surveyed species. In addition, we have shown that the core set of distinct conservation profiles is associated with a significant reduction in informational redundancy as compared to the complete set of profiles. Potentially, this reduction in the redundancy may reflect, more or less directly, reductions in the contributions of gene acquisition and loss processes in the evolutionary histories as captured by the profiles.

Beyond the descriptive analysis of profiles, we have also tried to assess problems and difficulties encountered in the derivation of trees from profiles. Thus, a reduction in informational redundancy, which may be an advantage in some respects, can also be too drastic if we consider the set of shared distinct profiles. Such stringent requirements, based on the normalized number of shared distinct conservation profiles between species, leads to the *minimal profiles tree* (Figure S1). However, the scheme retains only a very small percentage of the set of distinct conservation profiles, and much potentially significant information is discarded. We therefore opted here for a reasonable compromise, of calculating similarities between pairs of species from Jaccard scores based on the set of all distinct conservation profiles.

In short, the approach developed here is probably just a first step in treatment of the intrinsically multidimensional evolutionary histories of proteins to derive genome trees. Possibly, other data handling schemes may provide improved compromises between the criteria of maximal retention of relevant information and maximal removal of redundancies. For example, such improvements might derive from methods to calculate distances or similarity scores between species from conservation profiles, as well as measures of relatedness between species (for example, Manhattan, Euclidean, Chebyshev, and Hamming distances; see [59] for discussion).

Biologically, the results in the new *profiles tree* are better appreciated with a parallel analysis of three other trees (Figures S1, S2, and S3) obtained from identical genomic data. One major conclusion in this comparative analysis is the simultaneous observation of certain stable features and clusterings, along with clusterings that are highly variable following the trees (and the underlying methods of data analysis).

At the most general level, all the trees considered here display, invariably, a robust clustering of the studied species into three well defined groups corresponding to the three domains of life, as defined on the basis of 16/18S rRNA sequences [53]. Moreover, all the trees group the Archaea together with Bacteria. Such branching is consistent with the overall trend observed in various proteome comparisons that reveal Archaea are closer to Eukarya in terms of informational genes (transcription, translation) but closer to Bacteria for operational genes [9,60,61]. As all trees here are based on overall proteome comparisons, this very stable result adheres to a higher proportion of operational genes, rather than informational ones. This sibling relation is also consistent with universal trees, with artifacts due to long-branch attraction eliminated, in which Archaea are also clustered with Bacteria [62,63].

More detailed analysis reveals a series of prominent features in the three domains. Whatever the details of bacterial branch clustering, the Proteobacteria never form a homogeneous branch. Even so, within the bacterial branch certain associations are highly stable between trees such as the one which unites the parasitic Proteobacteria (Rickettsia species and in three trees the Buchnera species) with the Chlamydiae and the Spyrochaetes. A surprising example of variability is the position of the highly decayed *M. leprae* [64], which either clusters with the other Actinobacteria, or is separated from them to join *S. coelicolor* (with a highly expanded genome [65], separated from the other Actinobacteria in all cases). Similarly, the archaeal branch displays both stable and variable features such as the systematic clustering of *A. fulgidus* with Methanogens, and the variability of *Halobacterium* sp. which joins this cluster in only two of the trees. An even more striking example of variability is the location within this branch of the Pyrococcus cluster. Similarly we note in the eukaryal branch that the composite cluster {Plant-red algae-Protists} is linked with the Animals in all trees, but with the Fungi in the *conservation tree* (Figure S3).

Some unstable features observed in the various trees might potentially derive from a lack of adequate information (such as the number of representatives for given clades). Alternatively unstable features might originate in true evolutionary signals, such as dynamic features reshaping the genomes, toward either decays or expansions, and providing distinct versions, when analyzed with different schemes of data handling.

As discussed for several examples, differences between trees could account for a series of alternative phylogenetic hypotheses (monophyly of Methanogens, Coelomata versus Ecdysozoa, microsporidians with animals or fungi, etc). In such a perspective, several present controversies might then simply represent different facets of the same evolutionary reality. Possibly, the only reasonable road toward a global view of the genomic clustering of species would involve a combination of pictures from different trees. In addition, it seems important to keep track of the evolution of the variability features from different pictures, as the number of available genomes increases. Such information may tend to cause certain variabilities to recede or disappear while other (intrinsic variabilities) will remain independent of the number of representatives for the concerned clades. We have noted this tendency, in preliminary observations, as we have increased the number of genomes included in the present work from preliminary observations with smaller numbers of species. Those intrinsic variabilities, following the different points of view associated with the different types of analyses, may ultimately be preferentially associated with genome dynamics features. For such studies, we plan to update the various trees here (based on 99 species) as new data become available.

## Materials and Methods

### Species-specific comparisons.

The methodology for large-scale proteome comparisons (the list of species in the analysis is given in Table S1) has been described in detail elsewhere [23,66]. Briefly, the proteome of each species considered was compared to that of each other species (Figure 1, step 1), using the BLASTP program [67], with the pam250 substitution matrix and the *seg* filter [68]. The significance threshold for the comparisons was set heuristically for each target species. For example, probability score limits were set at 10^−9^ for all eukaryotic species (for details concerning *Saccharomyces cerevisiae* see [69]). From intra-proteome comparisons only reciprocal significant hits were retained, eliminating 2% to 5% of initial significant hits (with significant score in one comparison direction [A,B], and the score associated with the reciprocal direction [B,A] being non-significant). The results of all bidirectional pair-wise comparisons for the predicted proteomes (step 1 in Figure 1) permit the estimation of (a) the level of ancestral duplication in each species, (b) the ancestral conservation, (c) the number of shared orthologs between pairs of species (following the working definition of putative orthologs in [70]), and (d) the conservation profile for each protein across all considered species.

### Data tables and tree construction methods.

Figure 3 details the steps for the derivation of genome trees. For each data table considered, correspondence analysis [47,48] was used to plot species in a factorial space of dimensions *n*–1 (orthogonal system), with *n* the number of species. Species were then clustered according to their reciprocal neighborhood in the factorial space to obtain the genome tree. Correspondence analysis permits calculation of Euclidean distances using species coordinates in the factorial space.

The clustering process consists in grouping the two closest pairs of the *n *considered species (or terminal nodes), leading to (*n*–1) nodes. The two closest nodes among these (*n*–1) are then grouped to give (*n*–2) nodes, etc. This process is iterated (*n*–1) times until all species are grouped in a single node. The final tree shows the hierarchical clustering of all species in a decreasing order of neighborhood: closest species are clustered first and most distant last.

### Shared distinct conservation profiles.

The data matrix is defined as T = {T_ij_ = 100*s_ij_/s_jj_; i = 1, *n*; j = 1, *n*}, where T_ij_ represents the percent of shared distinct conservation profiles s_ij_ (see Figure 1, step 2) between species i and j relative to s_jj_, the total number of distinct conservation profiles in j. Note that among the total 184,130 distinct conservation profiles, only 24,044 are shared by at least two species, and the rest are unique to a given species. The corresponding tree is referred to as *minimal profiles tree.*

### Jaccard similarity scores between species.

In order to relax the strict restriction leading to the definition of shared conservation profiles and for taking into account the relevant ancestral information in the whole set of 184,130 distinct conservation profiles, we resort to similarity scores between species based on the Jaccard score. In this case the species are defined by binary vectors in the space of distinct conservation profiles.

The Jaccard score s_ij_ between two species i and j is calculated following the formula: s_ij_ = a_ij_/(a_ij_ + b_ij_ + c_ij_), where for indexes i and j (column indexes for the various species) the values of a_ij_, b_ij_, and c_ij_ are given, respectively, by the total number of occurrences of (1,1), (0,1), and (1,0) along the lines of the 184,130 distinct conservation profiles.

Following this definition, a Jaccard score varies between one (i and j are related at each line position of the distinct conservation profiles by a (1,1) pair) and zero (not a single (1,1) pair in all lines of the conservation profiles). As such, a Jaccard score can be considered as a normalized indicator of mutual conservation between pairs of species.

In this case the data matrix is defined as T = {T_ij_ = 100*s_ij_; i = 1, *n*; j = 1, *n*}, with T_ij_ expressing as a percentage the Jaccard score s_ij_ between species i and j. It can be noticed that the T matrix is symmetrical and that T_ii_ = 100 (since s_ii_ = 1). The tree derived from this data matrix is referred to as *profiles tree.*

### Partial trees associated with domains of life.

Two series of partial data tables were extracted from the previous table T, corresponding respectively to the bacterial, archaeal, and eukaryal domains. In the first series, restrictions concerned only the lines. For example, in such construction, the partial table associated with Eukaryotes is defined as T_E_ = {T_ij_ = 100*s_ij_; i = 1, p; j = 1, *n*}, where i is limited to the p eukaryotic species. The tree derived from this partial table is referred to as *eukarya subtree.* In the same way we define a *bacteria subtree* and an *archaea subtree.*

In the second series, the restrictions concerned the lines as well as the columns (lines and columns restricted to the species in a given domain). The trees derived from the corresponding data matrices are referred to as *bacteria only subtree, archaea only subtree,* and *eukarya only subtree,* respectively.

### Shared orthologs.

Two orthologous proteins are defined here as proteins with bidirectional best matches, in the comparison process. The central assumption in this approach is that orthologs display greater similarity to each other than to any other proteins from the respective genomes. The data matrix associated with shared orthologs is defined as T = {T_ij_ = 100*s_ij_/size(j); i = 1, *n;* j = 1, *n*}, where T_ij_ represents the percentage of shared orthologous proteins s_ij_ between species i and j, relatively to size(j), the total number of proteins in j. The tree derived from this data matrix is referred to as *orthologs tree.*

### Ancestral duplications and ancestral conservations.

The ancestral conservation s_ij_ is defined as the percentage of proteins in j that are conserved in i (i.e., proteins in j with at least one significant match in i), relative to the total number of proteins in j: s_ij_ = 100*(number of proteins in j that are conserved in i)/size(j). It can be noticed that for a given species j, s_jj_ corresponds to the weight of ancestral duplication. With this definition of the weights s_ij_, the ancestral duplication-conservation data matrix is: T = {T_i j_= s_ij_; i = 1, *n*; j = 1, *n*}. The tree derived from this data matrix is referred to as *conservation tree.* With the 99 genomes considered here, this tree corresponds in fact to an update of the tree derived previously from 15 genomes, as available in 1999 [23].

## Supporting Information

Figure S1 *Minimal Profiles Tree* Based on Shared Distinct Conservation Profiles See Materials and Methods.
(360 KB PDF)

Figure S2 *Orthologs Tree* Based on Shared Orthologs between Pairs of Species See Materials and Methods.
(340 KB PDF)

Figure S3 *Conservation Tree* Based on Ancestral Duplication and Ancestral Conservation Weights See Materials and Methods
(319 KB PDF)

Table S1 List of Predicted Proteomes Presented as They Appear in the Conservation Profiles and Corresponding References (94 KB DOC)
