# Supplementary material for: Genome Trees from Conservation Profiles
Source: PLoS Comput Biol. 2005 Dec 16;1(7):e75. doi: 10.1371/journal.pcbi.0010075 (PMC1314884; doi:10.1371/journal.pcbi.0010075)
Supplement: Supplementary file 1 [file pcbi.0010075.sg001.pdf]

S1

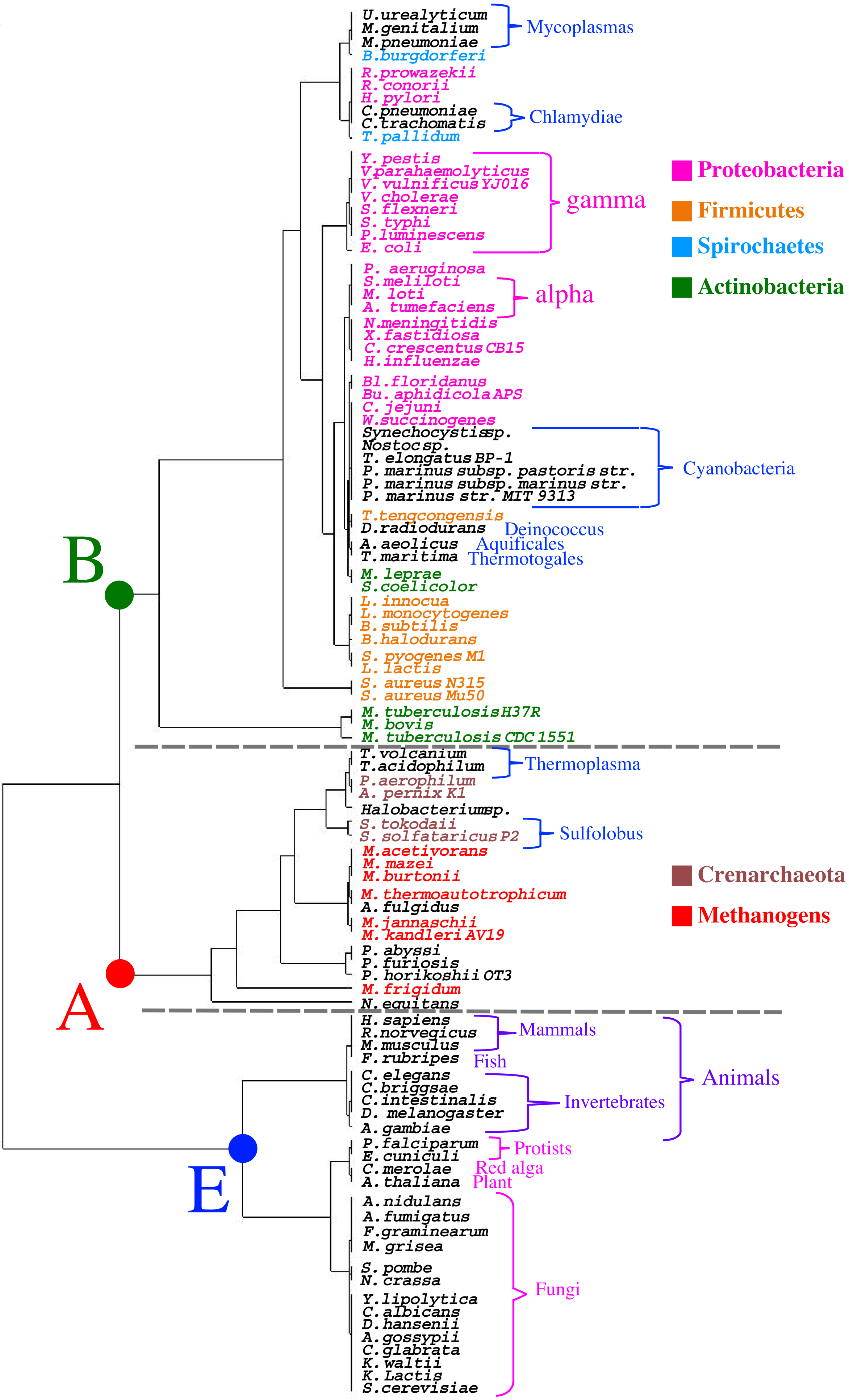

U.urealyticum
M.genitalium
M.pneumoniae
B.burgdorferi
Mycoplasmas
R.prowazekii
R.conorii
H.pylori
C.pneumoniae
C.trachomatis
T.pallidum
Chlamydiae
Y.pestis
V.parahaemolyticus
V.vulnificus YJ016
V.cholerae
S.flexneri
S.typhi
P.luminescens
E.coli
gamma
P.aeruginosa
S.meliloti
M.loti
A.tumefaciens
alpha
N.meningitidis
X.fastidiosa
C.crescentus CB15
H.influenzae
Bl.floridanus
Bu.aphidicola APS
C.jejuni
W.succinogenes
Synechocystis sp.
Nostoc sp.
T.elongatus BP-1
P.marinus subsp. pastoris str.
P.marinus subsp. marinus str.
P.marinus str. MIT 9313
Cyanobacteria
T.tengcongensis
D.radiodurans
Deinococcus
A.aeolicus
Aquificales
T.maritima
Thermotogales
M.leprae
S.coelicolor
L.innocua
L.monocytogenes
B.subtilis
B.halodurans
S.pyogenes M1
L.lactis
S.aureus N315
S.aureus Mu50
M.tuberculosis H37R
M.bovis
M.tuberculosis CDC 1551
Proteobacteria
Firmicutes
Spirochaetes
Actinobacteria
B
T.volcanium
T.acidophilum
Thermoplasma
P.aerophilum
A.pernix K1
Halobacterium sp.
S.tokodaii
S.solfataricus P2
Sulfolobus
M.acetivorans
M.mazei
M.burtonii
M.thermoautotrophicum
A.fulgidus
M.jannaschii
M.kandleri AV19
P.abyssi
P.furiosis
P.horikoshii OT3
M.frigidum
N.equitans
Crenarchaeota
Methanogens
A
H.sapiens
R.norvegicus
M.musculus
Mammals
F.rubripes
Fish
C.elegans
C.briggsae
C.intestinalis
D.melanogaster
A.gambiae
Invertebrates
Animals
P.falciparum
E.cuniculi
Protists
C.merolae
Red alga
A.thaliana
Plant
A.nidulans
A.fumigatus
F.graminearum
M.grisea
S.pombe
N.crassa
Y.lipolytica
C.albicans
D.hansenii
A.gossypii
C.glabrata
K.waltii
K.Lactis
S.cerevisiae
Fungi
E
