# Supplementary material for: Genome Trees from Conservation Profiles
Source: PLoS Comput Biol. 2005 Dec 16;1(7):e75. doi: 10.1371/journal.pcbi.0010075 (PMC1314884; doi:10.1371/journal.pcbi.0010075)
Supplement: Supplementary file 2 [file pcbi.0010075.sg002.pdf]

S2

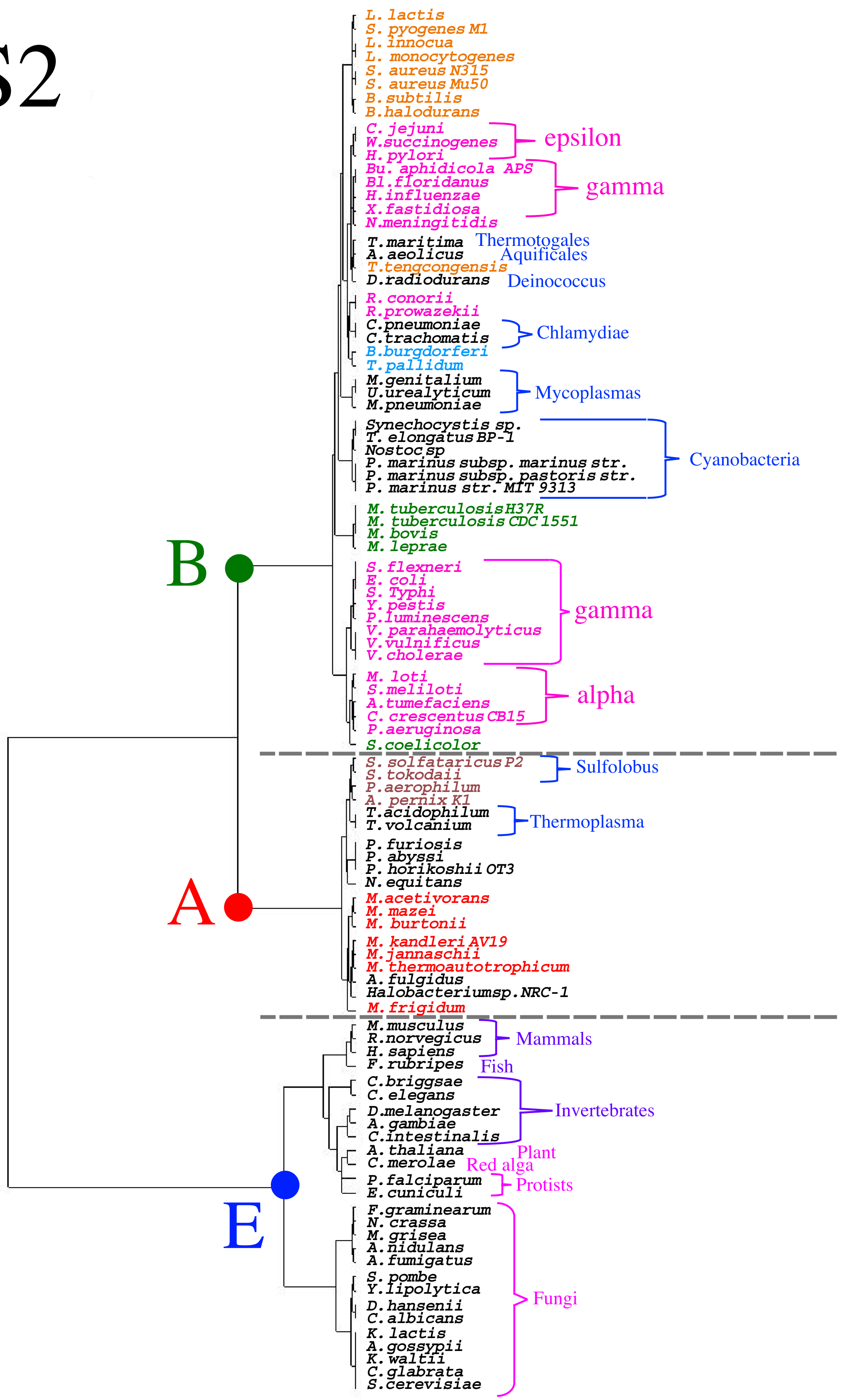

L. lactis
S. pyogenes M1
L. innocua
L. monocytogenes
S. aureus N315
S. aureus Mu50
B. subtilis
B. halodurans
C. jejuni
W. succinogenes
H. pylori
epsilon
Bu. aphidicola APS
Bl. floridanus
H. influenzae
X. fastidiosa
N. meningitidis
gamma
T. maritima
Thermotogales
A. aeolicus
Aquificales
T. tengcongensis
D. radiodurans
Deinococcus
R. conorii
R. prowazekii
C. pneumoniae
C. trachomatis
Chlamydiae
B. burgdorferi
T. pallidum
M. genitalium
U. urealyticum
M. pneumoniae
Mycoplasmas
Synechocystis sp.
T. elongatus BP-1
Nostoc sp
P. marinus subsp. marinus str.
P. marinus subsp. pastoris str.
P. marinus str. MIT 9313
Cyanobacteria
M. tuberculosis H37R
M. tuberculosis CDC 1551
M. bovis
M. leprae
B
S. flexneri
E. coli
S. Typhi
Y. pestis
P. luminescens
V. parahaemolyticus
V. vulnificus
V. cholerae
gamma
M. loti
S. meliloti
A. tumefaciens
C. crescentus CB15
P. aeruginosa
alpha
S. coelicolor
S. solfataricus P2
S. tokodaii
Sulfolobus
P. aerophilum
A. pernix K1
T. acidophilum
T. volcanium
Thermoplasma
P. furiosis
P. abyssi
P. horikoshii OT3
N. equitans
A
M. acetivorans
M. mazei
M. burtonii
M. kandleri AV19
M. jannaschii
M. thermoautotrophicum
A. fulgidus
Halobacterium sp. NRC-1
M. frigidum
M. musculus
R. norvegicus
H. sapiens
Mammals
F. rubripes
Fish
C. briggsae
C. elegans
D. melanogaster
A. gambiae
C. intestinalis
Invertebrates
A. thaliana
Plant
C. merolae
Red alga
P. falciparum
E. cuniculi
Protists
E
F. graminearum
N. crassa
M. grisea
A. nidulans
A. fumigatus
S. pombe
Y. lipolytica
D. hansenii
C. albicans
K. lactis
A. gossypii
K. waltii
C. glabrata
S. cerevisiae
Fungi
