# Supplementary material for: Genome Trees from Conservation Profiles
Source: PLoS Comput Biol. 2005 Dec 16;1(7):e75. doi: 10.1371/journal.pcbi.0010075 (PMC1314884; doi:10.1371/journal.pcbi.0010075)
Supplement: Supplementary file 3 [file pcbi.0010075.sg003.pdf]

S3

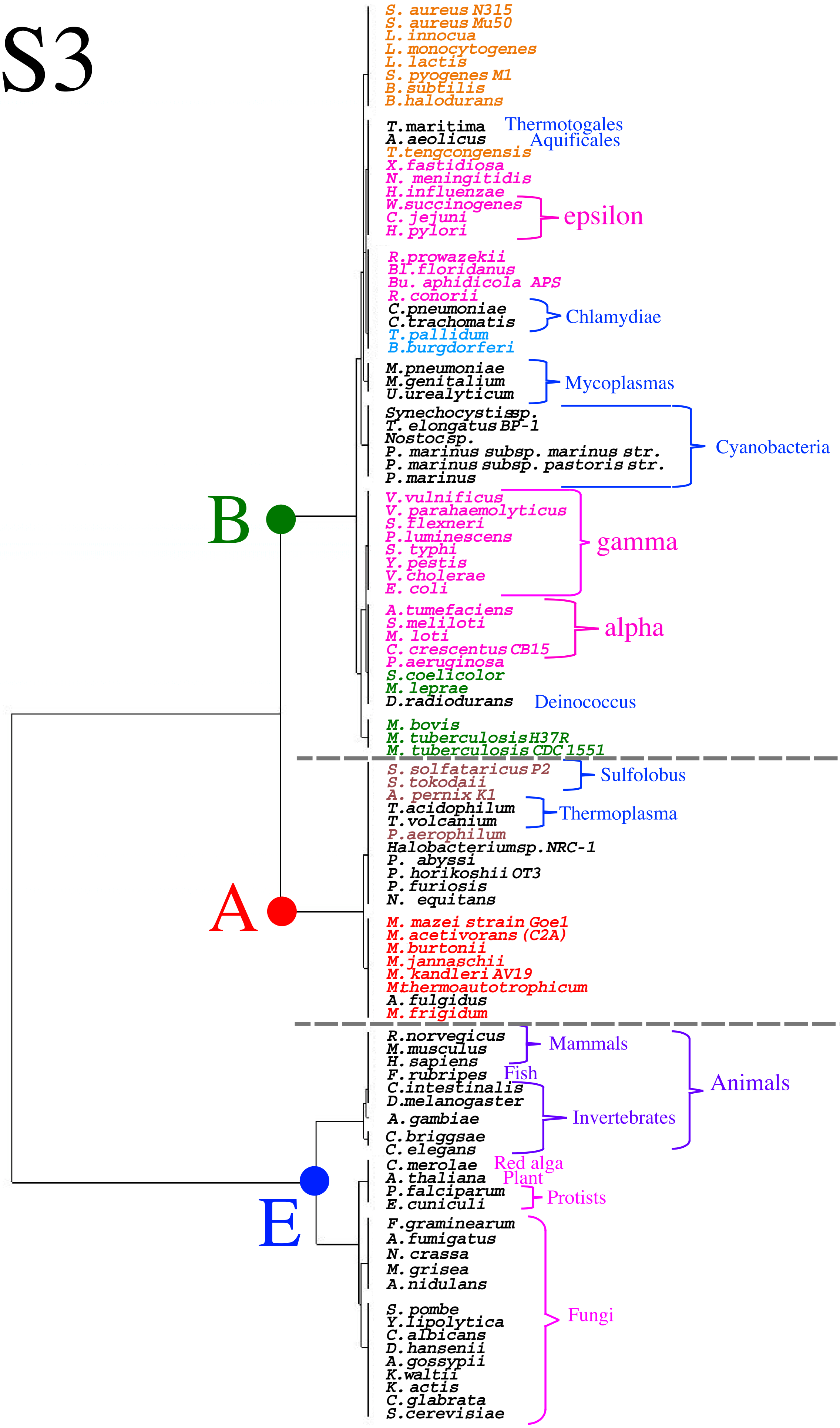

S. aureus N315
S. aureus Mu50
L. innocua
L. monocytogenes
L. lactis
S. pyogenes M1
B. subtilis
B.halodurans
T.maritima
Thermotogales
A.aeolicus
Aquificales
T.tengcongensis
X.fastidiosa
N. meningitidis
H.influenzae
W.succinogenes
C. jejuni
H. pylori
epsilon
R.prowazekii
Bl.floridanus
Bu. aphidicola APS
R. conorii
C.pneumoniae
C.trachomatis
Chlamydiae
T.pallidum
B.burgdorferi
M.pneumoniae
M.genitalium
U.urealyticum
Mycoplasmas
Synechocystis sp.
T. elongatus BP-1
Nostoc sp.
P. marinus subsp. marinus str.
P. marinus subsp. pastoris str.
P.marinus
Cyanobacteria
V.vulnificus
V. parahaemolyticus
S. flexneri
P.luminescens
S. typhi
Y. pestis
V. cholerae
E. coli
gamma
A.tumefaciens
S.meliloti
M. loti
C. crescentus CB15
P.aeruginosa
alpha
S.coelicolor
M. leprae
D.radiodurans
Deinococcus
M. bovis
M. tuberculosis H37R
M. tuberculosis CDC 1551
B
S. solfataricus P2
S. tokodaii
Sulfolobus
A. pernix K1
T.acidophilum
T.volcanium
Thermoplasma
P.aerophilum
Halobacterium sp. NRC-1
P. abyssi
P. horikoshii OT3
P. furiosis
N. equitans
M. mazei strain Goe1
M. acetivorans (C2A)
M.burtonii
M.jannaschii
M. kandleri AV19
M thermoautotrophicum
A. fulgidus
M. frigidum
A
R.norvegicus
M.musculus
H. sapiens
Mammals
F. rubripes
Fish
C.intestinalis
D.melanogaster
A. gambiae
C.briggsae
C. elegans
Invertebrates
Animals
C. merolae
Red alga
A. thaliana
Plant
P.falciparum
E.cuniculi
Protists
F.graminearum
A.fumigatus
N. crassa
M. grisea
A.nidulans
S. pombe
Y.lipolytica
C.albicans
D.hansenii
A.gossypii
K.waltii
K. actis
C.glabrata
S.cerevisiae
Fungi
E
