# Supplementary material for: Genome Trees from Conservation Profiles
Source: PLoS Comput Biol. 2005 Dec 16;1(7):e75. doi: 10.1371/journal.pcbi.0010075 (PMC1314884; doi:10.1371/journal.pcbi.0010075)
Supplement: Supplementary file 4 [file pcbi.0010075.st001.doc]

**Table S1: Predicted proteome data, presented as they appear in the conservation profiles.**

We considered a set of 99 completely or almost completely sequenced species representing a significant diversity: 27 eucaryotes, 19 archaeal and 53 bacterial species, totalling 541880 proteins. Species names, their corresponding total predicted ORF products and codes are listed below as appearing in the conservation profiles (Figure 1). Data were downloaded from species specific server when available or from the ncbi ftp server ([ftp.ncbi.nih.gov/genomes/](ftp://ftp.ncbi.nih.gov/genomes/)).

*Eukarial* predicted proteomes (27) totalling 344272 proteins: *Saccharomyces cerevisiae* (5829; [1]), *Schizosaccharomyces pombe* (4962; [2]), *Neurospora crassa* (10082; [3]), *Candida albicans* (6114; [4, 5]), *Magnaporthe Grisea* (11109; [6]), *Fusarium Graminearum* (11640; [7]), *Aspergillus nidulans* (9541; [8]),  *Encephalitozoon cuniculi* (1996; [9]), *Caenorhabditis elegans* (20844; [10]), *Caenorhabditis briggsae* (25111; [11]), *Drosophila melanogaster* (17878; [12]), *Anopheles gambiae* (16112; [13]), *Arabidopsis thaliana* (22671; [14]), *Homo sapiens* (27625, ncbi version available on March 2003; [15]), *Mus musculus* (28099; [16]), *Rattus norvegicus* (21205; [17]), *Fugu rubripes* (33609; [18]), *Plasmodium falciparum* (5334; [19]), *Ciona intestinalis* (15851; [20]), *Aspergilus fumigatus* (9746; TIGR: http://www.tigr.org/tdb/e2k1/afu1/), *Ashbya gossypii* (4718; [21]), *Candida glabrata* (5275; [22]), *Yarrowia lipolytica* (6666; [22]), *Debaryomyces hansenii* (6937; [22]), *Kluyveromyces Lactis* (5334; [22]), *Kluyveromyces waltii* (5214; [23]) and *Cyanidioschyzon merolae* (4772; [24]).

*Archaeal* predicted proteomes (19) totalling 42062 proteins: *Methanococcus jannaschii* ( 1773; [25]), *Methanobacterium thermoautotrophicum* (1871; [26]), *Archaeoglobus fulgidus* (2409; [27]), *Pyrococcus horikoshii OT3* (2061; [28]), *Pyrococcus abyssi* (1765; [29]), *Aeropyrum pernix* K1 (1865, [30]), *Thermoplasma acidophilum* (1478; [31]), *Thermoplasma volcanium* (1526; [32]), *Halobacterium sp.* NRC-1 (2058; [33]), *Sulfolobus solfataricus* P2 (2977; [34]), *Pyrococcus furiosis* (2208; [35]), *Sulfolobus tokodaii* (2826; [36]), *Pyrobaculum aerophilum* (2605; [37]), *Methanosarcina acetivorans* (C2A) (4528; [38]), *Methanopyrus kandleri* AV19 (1687; [39]), *Methanosarcina mazei* strain Goe1 (3371; [40]), *Methanococcoides burtonii* (2676; [41]), *Methanogenium frigidum* (1815; [41]) and *Nanoarchaeum equitans* (563; [42]).

*Bacterial* predicted proteomes (53) totalling 155546 proteins: *Haemophilus influenzae* (1713; [43]), *Mycoplasma genitalium* (479; [44]), *Mycoplasma pneumoniae* (677; [45]), *Synechocystis sp.* (3168; [46]), *Escherichia coli* (4290; [47]), *Helicobacter pylori* (1577; [48]), *Bacillus subtilis* (4100; [49]), *Bacillus halodurans* (4066; [50]), *Borrelia burgdorferi* (1639; [51]), *Aquifex aeolicus* (1522; [52]), *Mycobacterium tuberculosis* H37R (3996; [53]), *M. tuberculosis* CDC 1551 (4203; [54]), *Mycobacterium leprae* (1604; [55]), *Treponema pallidum* (1031; [56]), *Chlamydia trachomatis* (877; [57]), *Rickettsia prowazekii* (837; [58]), *Campylobacter jejuni* (1634; [59]), *Chlamydia pneumoniae* (1052; [60]), *Thermotoga maritima* (1849; [61]), *Deinococcus radiodurans* (3117; [62]), *Neisseria meningitidis* (NM, 2081; [63]), *Xylella fastidiosa* (2830; [64]), *Vibrio cholerae* (3837; [65]), *Pseudomonas aeruginosa* (5570; [66]), *Buchnera sp.* (575; [67]), *Listeria monocytogenes* EGD-e (2846; [68]), *Listeria innocua* (2968; [68]), *Salmonella Typhi* (4395; [69]), *Yersinia pestis* (3895; [70]), *Staphylococcus aureus* Mu50 (2714; [71]), *Staphylococcus aureus* N315 (2594; [71]), *Streptococcus pyogenes* M1 (1696; [72]), *Mesorhizobium loti* (7275; [73]), *Sinorhizobium meliloti* (6205; [74]), *Agrobacterium tumefaciens* (5299; [75]), *Mycobacterium Bovis* ( 3953; [76]), *Streptomyces coelicolor* (7810; [77]), *Ureaplasma urealyticum* (614; [78]), *Shigella flexneri* (4068; [79]), *Lactococcus lactis subsp. lactis* (2321; [80]), *Rickettsia conorii* Malish 7 (1374; [81]), *Caulobacter crescentus* CB15 (3737; [82]), *Nostoc* sp. (5366; [83]), *Thermosynechococcus elongatus BP-1* (2475; [84]), *Thermoanaerobacter tengcongensis strain MB4T* (2588; [85]), *Candidatus Blochmannia floridanus* (583; [86]), *Prochlorococcus marinus subsp. marinus str.* CCMP1375 (1882; [87]), *Prochlorococcus marinus str. MIT 9313* (2265; [88]), *Prochlorococcus marinus subsp. pastoris str.* CCMP1378 (1712; [88]), *Wolinella succinogenes* (2044; [89]), *Photorhabdus luminescens subsp. laumondii* (4683; [90]), *Vibrio parahaemolyticus RIMD 2210633* (4832; [91]) and *Vibrio vulnificus YJ016* (VVYJ, 5028; [92]).

**Complete genome references (supplementary table S1):**

1. Goffeau A, Barrell BG, Bussey H, Davis RW, Dujon B, Feldmann H, Galibert F, Hoheisel JD, Jacq C, Johnston M, Louis EJ, Mewes HW, Murakami Y, Philippsen P, Tettelin H, Oliver SG (1996) Life with 6000 genes. Science 274, 563–567.

2. Wood V, Gwilliam R, Rajandream MA, Lyne M, Lyne R, Stewart A, Sgouros J, Peat N, Hayles J, Baker S, et al. (2002) The genome sequence of *Schizosaccharomyces pombe*. Nature 415:871-80.

3. Galagan JE, Calvo SE, Borkovich KA, Selker EU, Read ND, Jaffe D, FitzHugh W, Ma LJ, Smirnov S, Purcell S, et al. (2003) The genome sequence of the filamentous fungus *Neurospora crassa*. Nature 422:859-68.

4. d'Enfert C, Goyard S, Rodriguez-Arnaveilhe S, Frangeul L, Jones L, Tekaia F, Bader O, Antje Albrecht, Castillo L, Dominguez A, et al. (2005) CandidaDB: a genomic database for *Candida albicans* pathogenomics. Nucl. Acid Res. (Database issue): D353-7.

5. Jones T, Federspiel NA, Chibana H, Dungan J, Kalman S, Magee BB, Newport G, Thorstenson YR, Agabian N, Magee PT, et al. (2004) The diploid genome sequence of *Candida albicans*. Proc Natl Acad Sci U S A.101:7329-7334.

6. Dean RA, Talbot NJ, Ebbole DJ, Farman ML, Mitchell TK, et al. (2005) The genome sequence of the rice blast fungus *Magnaporthe grisea.* Nature 434, 980-986.

And Broad Institute; http://www.broad.mit.edu/annotation/fungi/magnaporthe/

7. Broad Institute: <http://www.broad.mit.edu/annotation/fungi/fusarium/>

8. Broad Institute: <http://www.broad.mit.edu/annotation/fungi/aspergillus/>

9. Katinka MD, Duprat S, Cornillot E, Metenier G, Thomarat F, Prensier G, Barbe V, Peyretaillade E, Brottier P, Wincker P, et al. (2001) Genome sequence and gene compaction of the eukaryote parasite *Encephalitozoon cuniculi*. Nature 414:450-3.

10. The *C. elegans* Sequencing Consortium. (1998) Genome sequence of the nematode *Caenorhabditis elegans*: A platform for investigating biology. Science 282: 2012-2018.

11. Stein LD, Bao Z, Blasiar D, Blumenthal T, Brent MR, Chen N, Chinwalla A, Clarke L, Clee C, Coghlan A, et al. (2003) The Genome Sequence of *Caenorhabditis briggsae*: A Platform for Comparative Genomics. PLoS Biol.1:E45.

12. Adams MD, Celniker SE, Holt RA, Evans CA, Gocayne JD, Amanatides PG, Scherer SE, Li PW, Hoskins RA, Galle RF et al. (2000) The genome sequence of *Drosophila melanogaster*. Science 287:2185-95.

13. Holt RA, Subramanian GM, Halpern A, Sutton GG, Charlab R, Nusskern DR, Wincker P, Clark AG, Ribeiro JM, Wides R, et al. (2002) The genome sequence of the malaria mosquito *Anopheles gambiae*. Science 298:129-49.

14. Arabidopsis Genome Initiative. (2000) Analysis of the genome sequence of the flowering plant Arabidopsis thaliana. Nature 408:796-815.

15. Lander ES, Linton LM, Birren B, Nusbaum C, Zody MC, Baldwin J, Devon K, Dewar K, Doyle M, FitzHugh W, et al. (2001) Initial sequencing and analysis of the human genome. Nature 409:860-921.

16. Waterston RH, Lindblad-Toh K, Birney E, Rogers J, Abril JF, Agarwal P, Agarwala R, Ainscough R, Alexandersson M, An P, Antonarakis SE, et al. (2002) Initial sequencing and comparative analysis of the mouse genome. Nature 420:520-62.

17. Rat Genome Sequencing Project Consortium. (2004) Genome sequence of the Brown Norway rat yields insights into mammalian evolution. Nature 428, 493 - 521.

18. Aparicio S, Chapman J, Stupka E, Putnam N, Chia JM, Dehal P, Christoffels A, Rash S, Hoon S, Smit A, et al. ((2002) Whole-genome shotgun assembly and analysis of the genome of *Fugu rubripes*. Science 297:1301-10.

19. Gardner MJ, Hall N, Fung E, White O, Berriman M, Hyman RW, Carlton JM, Pain A, Nelson KE, Bowman S, et al. (2002) Genome sequence of the human malaria parasite *Plasmodium falciparum*. Nature 419:498-511.

20. Dehal P, Satou Y, Campbell RK, Chapman J, Degnan B, De Tomaso A, Davidson B, Di Gregorio A, Gelpke M, Goodstein DM, et al. (2002) The draft genome of *Ciona intestinalis*: insights into chordate and vertebrate origins. Science 298:2157-67.

21. Dietrich FS, Voegeli S, Brachat S, Lerch A, Gates K, Steiner S, Mohr C, Pohlmann R, Luedi P, Choi S, et al. (2004) The *Ashbya gossypii* genome as a tool for mapping the ancient Saccharomyces cerevisiae genome. Science 304:304-7.

22. Dujon B, Sherman D, Fischer G, Durrens P, Casaregola S, Lafontaine I, De Montigny J, Marck C, Neuveglise C, Talla E, et al. (2004) Genome evolution in yeasts. Nature 430: 35-44.

23. Kellis M, Birren BW, Lander ES (2004) Proof and evolutionary analysis of ancient genome duplication in the yeast *Saccharomyces cerevisiae*. Nature 428:617-24.

24. Ohta N, Matsuzaki M, Misumi O, Miyagishima SY, Nozaki H, Tanaka K, Shin-I T, Kohara Y and Kuroiwa T (2003) Complete sequence and analysis of the plastid genome of the unicellular red alga *Cyanidioschyzon merolae.* DNA Res. 10:67-77.

25. Bult CJ, White O, Olsen GJ, Zhou L, Fleischmann RD, Sutton GG, Blake JA, FitzGerald LM, Clayton RA, Gocayne JD (1996) Complete genome sequence of the methanogenic archaeon, *Methanococcus jannaschii.* Science 273:1058-1073

26. Smith DR, Doucette-Stamm LA, Deloughery C, Lee H, Dubois J, Aldredge T, Bashirzadeh R, Blakely D, Cook R, Gilbert K (1997) Complete genome sequence of *Methanobacterium thermoautotrophicum* deltaH: Functional analysis and comparative genomics. J. Bacteriol. 179: 7135-7155.

27. Klenk HP, Clayton RA, Tomb JF, White O, Nelson KE, Ketchum KA, Dodson RJ, Gwinn M, Hickey EK, Peterson JD (1997) The complete genome sequence of the hyperthermophilic, sulphate-reducing archaeon *Archaeoglobus fulgidus.* Nature 390: 364-370.

28. Kawarabayasi Y, Sawada M, Horikawa H, Haikawa Y, Hino Y, Yamamoto S, Sekine M, Baba S, Kosugi H, Hosoyama A (1998) Complete sequence and gene organization of the genome of a hyper-thermophilic archaebacterium, *Pyrococcus horikoshii* OT3. DNA Res. 5: 55-76.

29. Cohen GN, Barbe V, Flament D, Galperin M, Heilig R, Lecompte O, Poch O, Prieur D, Querellou J, Ripp R, et al. (2003) An integrated analysis of the genome of the hyperthermophilic archaeon *Pyrococcus abyssi*. Mol. Microbiol. 47:1495-1512.

30. Natale DA, Shankavaram UT, Galperin MY, Wolf YI, Aravind L, Koonin EV (2000) Towards understanding the first genome sequence of a crenarchaeon by genome annotation using clusters of orthologous groups of proteins (COGs). Genome Biol. 5: 1-19.

31. Ruepp A, Graml W, Santos-Martinez ML, Koretke KK, Volker C, Mewes HW, Frishman D, Stocker S, Lupas AN, Baumeister W (2000) The genome sequence of the thermoacidophilic scavenger *Thermoplasma acidophilum*. Nature 407:508-13.

32. Kawashima T, Amano N, Koike H, Makino S, Higuchi S, Kawashima-Ohya Y, Watanabe K, Yamazaki M, Kanehori K, Kawamoto T, et al. (2000) Archaeal adaptation to higher temperatures revealed by genomic sequence of *Thermoplasma volcanium*. Proc Natl Acad Sci U S A. 97:14257-62.

33. Ng WV, Kennedy SP, Mahairas GG, Berquist B, Pan M, Shukla HD, Lasky SR, Baliga NS, Thorsson V, Sbrogna J, et al. (2000) Genome sequence of *Halobacterium* species NRC-1. Proc Natl Acad Sci U S A 97:12176-81.

34. She Q, Singh RK, Confalonieri F, Zivanovic Y, Allard G, Awayez MJ, Chan-Weiher CC, Clausen IG, Curtis BA, De Moors A, et al. (2001) The complete genome of the crenarchaeon *Sulfolobus solfataricus* P2. Proc Natl Acad Sci U S A. 98:7835-40.

35. Robb FT, Maeder DL, Brown JR, DiRuggiero J, Stump MD, Yeh RK, Weiss RB, Dunn DM (2001) Genomic sequence of hyperthermophile, *Pyrococcus furiosus*: implications for physiology and enzymology. Methods Enzymol 330:134-57.

36. Kawarabayasi Y, Hino Y, Horikawa H, Jin-no K, Takahashi M, Sekine M, Baba S, Ankai A, Kosugi H, Hosoyama A, et al. (2001) Complete genome sequence of an aerobic thermoacidophilic crenarchaeon, *Sulfolobus tokodaii* strain7. DNA Res. 8:123-40.

37. Fitz-Gibbon ST, Ladner H, Kim UJ, Stetter KO, Simon MI, Miller JH (2002) Genome sequence of the hyperthermophilic crenarchaeon *Pyrobaculum aerophilum*. Proc Natl Acad Sci U S A. 99:984-9.

38. Galagan JE, Nusbaum C, Roy A, Endrizzi MG, Macdonald P, FitzHugh W, Calvo S, Engels R, Smirnov S, Atnoor D, et al. (2002) The genome of *M. acetivorans* reveals extensive metabolic and physiological diversity. Genome Res.12:532-42.

39. Slesarev AI, Mezhevaya KV, Makarova KS, Polushin NN, Shcherbinina OV, Shakhova VV, Belova GI, Aravind L, Natale DA, Rogozin IB, et al. (2002) The complete genome of hyperthermophile *Methanopyrus kandleri* AV19 and monophyly of archaeal methanogens. Proc Natl Acad Sci U S A. 99:4644-9.

40. Deppenmeier U, Johann A, Hartsch T, Merkl R, Schmitz RA, Martinez-Arias R, Henne A, Wiezer A, Baumer S, Jacobi C, et al. (2002) The genome of *Methanosarcina mazei*: evidence for lateral gene transfer between bacteria and archaea. J Mol Microbiol Biotechnol. 4:453-61.

41. Saunders NF, Thomas T, Curmi PM, Mattick JS, Kuczek E, Slade R, Davis J, Franzmann PD, Boone D, Rusterholtz K, et al. (2003) Mechanisms of thermal adaptation revealed from the genomes of the Antarctic Archaea *Methanogenium frigidum* and *Methanococcoides burtonii*. Genome Res.13:1580-8.

42. Waters E, Hohn MJ, Ahel I, Graham DE, Adams MD, Barnstead M, Beeson KY, Bibbs L, Bolanos R, Keller M, et al. (2003) The genome of *Nanoarchaeum equitans*: insights into early archaeal evolution and derived parasitism. Proc Natl Acad Sci U S A.100:12984-8.

43. Fleischmann RD, Adams MD, White O, Clayton RA, Kirkness EF, Kerlavage AR, Bult CJ, Tomb J-F, Dougherty BA, Merrick JM (1995) Whole-genome random sequencing and assembly of *Haemophilus influenzae* Rd. Science 269:496-512.

44. Fraser CM, Gocayne JD, White O, Adams MD, Clayton RA, Fleischmann RD, Bult CJ, Kerlavage AR, Sutton G, Kelley JM (1995) The minimal gene complement of *Mycoplasma genitalium.* Science 270: 397-403.

45. Himmelreich R, Hilbert H, Plagens H, Pirkl E, Li B-C, and Herrmann R (1996) Complete sequence analysis of the genome of the bacterium *Mycoplasma pneumoniae.* Nucleic Acid Res. 24: 4420-4449.

46. Kaneko T, Sato S, Kotani H, Tanaka A, Asamizu E, Nakamura Y, Miyajima N, Hirosawa M, Sugiura M, Sasamoto S (1996) Sequence analysis of the genome of the unicellular *cyanobacterium Synechocystis* sp. strain PCC6803. II. Sequence determination of the entire genome and assignment of potential protein-coding regions. *DNA Res. 3: 109-136.*

47. Blattner FR, Plunkett G III, Bloch CA, Perna NT, Burland V, Riley M, Collado-Vides J, Glasner JD, Rode CK, Mayhew GF (1997) The complete genome sequence of *Escherichia coli* K-12. Science 277:1453-1462.

48. Tomb JF, White O, Kerlavage AR, Clayton RA, Sutton GG, Fleischmann RD, Ketchum KA, Klenk HP, Gill S, and Dougherty BA (1997) The complete genome sequence of the gastric pathogen *Helicobacter pylori.* Nature 388:539-547.

49. Kunst F, Ogasawara N, Moszer I, Albertini AM, Alloni G, Azevedo V, Bertero MG, Bessieres P, Bologin A, Borchert S (1997) The complete genome sequence of the Grampositive bacterium *Bacillus subtilis.* Nature 390: 249-259.

50. Takami H, Horikoshi K (2000) Analysis of the genome of an alkaliphilic Bacillus strain from an industrial point of view. Extremophiles 4:99-108.

51. Fraser CM, Casjens S, Huang WM, Sutton GG, Clayton R, Lathigra R, White O, Ketchum KA, Dodson R, Hickey EK (1997) Genomic sequence of a Lyme disease spirochaete, *Borrelia burgdorferi.* Nature 390: 580-586.

52. Deckert G, Warren PV, Gaasterland T, Young WG, Lenox AL, Graham DE, Overbeek R, Snead MA, Keller M, Aujay M (1998) The complete genome of the hyperthermophilic bacterium *Aquifex aeolicus*. Nature 392: 353-358.

53. Cole ST, Brosch R, Parkhill J, Garnier T, Churcher C, Harris D, Gordon SV, Eiglmeier K, Gas S, Barry CE 3rd, Tekaia F, et al. (1998) Deciphering the biology of *Mycobacterium tuberculosis* from the complete genome sequence. Nature 393:537-544.

54. Fleischmann RD, Alland D, Eisen JA, Carpenter L, White O, Peterson J, DeBoy R, Dodson R, Gwinn M, Haft D, et al. (2002) Whole-genome comparison of *Mycobacterium tuberculosis* clinical and laboratory strains. J Bacteriol.184:5479-90.

55. Cole ST, Eiglmeier K, Parkhill J, James KD, Thomson NR, Wheeler PR, Honore N, Garnier T, Churcher C, Harris D et al. (2001) Massive gene decay in the leprosy bacillus. Nature 409:1007-1011.

56. Fraser CM, Norris SJ, Weinstock GM, White O, Sutton GG, Dodson R, Gwinn M, Hickey EK, Clayton R, Ketchum KA (1998) Complete genome sequence of *Treponema pallidum*, the syphilis spirochete. Science 281: 375-388.

57. Stephens RS, Kalman S, Lammel C, Fan J, Marathe R, Aravind L, Mitchell W, Olinger L, Tatusov RL, Zhao Q, et al. (1998) Genome sequence of an obligate intracellular pathogen of humans: *Chlamydia trachomatis*. Science 282:754-9.

58. Andersson SG, Zomorodipour A, Andersson JO, Sicheritz-Ponten T, Alsmark UC, Podowski RM, Naslund AK, Eriksson AS, Winkler HH, Kurland CG (1998) The genome sequence of *Rickettsia prowazekii* and the origin of mitochondria. Nature 396: 133-140.

59. Parkhill J, Wren BW, Mungall K, Ketley JM, Churcher C, Basham D, Chillingworth T, Davies RM, Feltwell T, Holroyd S, et al. (2000) The genome sequence of the food-borne pathogen *Campylobacter jejuni* reveals hypervariable sequences. Nature 403:665-8.

60. Kalman S, Mitchell W, Marathe R, Lammel C, Fan J, Hyman RW, Olinger L, Grimwood J, Davis RW and Stephens RS (1999) Comparative genomes of *Chlamydia pneumoniae* and *C. trachomatis*. Nat. Genet. 21, 385-389.

61. Nelson KE, Clayton RA, Gill SR, Gwinn ML, Dodson RJ, Haft DH, Hickey EK, Peterson JD, Nelson WC, Ketchum KA, et al. (1999) Evidence for lateral gene transfer between Archaea and bacteria from genome sequence of *Thermotoga maritima*. Nature 399:323-9.

62. White O, Eisen JA, Heidelberg JF, Hickey EK, Peterson JD, Dodson RJ, Haft DH, Gwinn ML, Nelson WC, Richardson DL, et al. (1999) Genome sequence of the radioresistant bacterium *Deinococcus radiodurans* R1. Science 286:1571-7.

63. Tettelin H, Saunders NJ, Heidelberg J, Jeffries AC, Nelson KE, Eisen JA, Ketchum KA, Hood DW, Peden JF, Dodson RJ, et al. (2000) Complete genome sequence of Neisseria *meningitidis serogroup* B strain MC58. Science 287:1809-15.

64. Simpson AJ, Reinach FC, Arruda P, Abreu FA, Acencio M, Alvarenga R, Alves LM, Araya JE, Baia GS, Baptista CS, et al. (2000) The genome sequence of the plant pathogen *Xylella fastidiosa*. The *Xylella fastidiosa* Consortium of the Organization for Nucleotide Sequencing and Analysis. Nature 406:151-7.

65. Heidelberg JF, Eisen JA, Nelson WC, Clayton RA, Gwinn ML, Dodson RJ, Haft DH, Hickey EK, Peterson JD, Umayam L, et al. (2000) DNA sequence of both chromosomes of the cholera pathogen *Vibrio cholerae*. Nature 406:477-83.

66. Stover CK, Pham XQ, Erwin AL, Mizoguchi SD, Warrener P, Hickey MJ, Brinkman FS, Hufnagle WO, Kowalik DJ, Lagrou M, et al. (2000) Complete genome sequence of *Pseudomonas aeruginosa* PA01, an opportunistic pathogen. Nature 406:959-64.

67. Shigenobu S, Watanabe H, Hattori M, Sakaki Y, Ishikawa H (2000) Genome sequence of the endocellular bacterial symbiont of aphids *Buchnera sp*. APS. Nature 407:81-6.

68. Glaser P, Frangeul L, Buchrieser C, Rusniok C, Amend A, Baquero F, Berche P, Bloecker H, Brandt P, Chakraborty T, et al. (2001) Comparative genomics of *Listeria* species. Science 294:849-52.

69. Parkhill J, Dougan G, James KD, Thomson NR, Pickard D, Wain J, Churcher C, Mungall KL, Bentley SD, Holden MT, et al. (2001) Complete genome sequence of a multiple drug resistant *Salmonella enterica serovar Typhi* CT18. Nature 413:848-52.

70. Parkhill J, Wren BW, Thomson NR, Titball RW, Holden MT, Prentice MB, Sebaihia M, James KD, Churcher C, Mungall KL, et al. (2001) Genome sequence of *Yersinia pestis*, the causative agent of plague. Nature 413:523-7.

71. Kuroda M, Ohta T, Uchiyama I, Baba T, Yuzawa H, Kobayashi I, Cui L, Oguchi A, Aoki K, Nagai Y, et al. (2001) Whole genome sequencing of meticillin-resistant *Staphylococcus aureus*. Lancet 357:1225-40.

72. Ferretti JJ, McShan WM, Ajdic D, Savic DJ, Savic G, Lyon K, Primeaux C, Sezate S, Suvorov AN, Kenton S, et al. (2001) Complete genome sequence of an M1 strain of *Streptococcus pyogenes*. Proc Natl Acad Sci U S A. 98:4658-63.

73. Kaneko T, Nakamura Y, Sato S, Asamizu E, Kato T, Sasamoto S, Watanabe A, Idesawa K, Ishikawa A, Kawashima K, et al. (2000) Complete genome structure of the nitrogen-fixing symbiotic bacterium *Mesorhizobium loti*. DNA Res. 7:331-8.

74. Galibert F, Finan TM, Long SR, Puhler A, Abola P, Ampe F, Barloy-Hubler F, Barnett MJ, Becker A, Boistard P, et al. (2001) The composite genome of the legume symbiont *Sinorhizobium meliloti*. Science 293:668-72.

75. Goodner B, Hinkle G, Gattung S, Miller N, Blanchard M, Qurollo B, Goldman BS, Cao Y, Askenazi M, Halling C, et el. (2001) Genome sequence of the plant pathogen and biotechnology agent *Agrobacterium tumefaciens* C58. Science 294:2323-8.

76. Garnier T, Eiglmeier K, Camus JC, Medina N, Mansoor H, Pryor M, Duthoy S, Grondin S, Lacroix C, Monsempe C, et al. (2003) The complete genome sequence of *Mycobacterium bovis*. Proc Natl Acad Sci U S A. 100:7877-82.

77. Bentley SD, Chater KF, Cerdeno-Tarraga AM, Challis GL, Thomson NR, James KD, Harris DE, Quail MA, Kieser, H, Harper D, et al. (2002) Complete genome sequence of the model actinomycete *Streptomyces coelicolor* A3(2). Nature 417:141-7.

78. Glass JI, Lefkowitz EJ, Glass JS, Heiner CR, Chen EY, Cassell GH. (2000) The complete sequence of the mucosal pathogen *Ureaplasma urealyticum*. Nature 407:757-62.

79. Wei J, Goldberg MB, Burland V, Venkatesan MM, Deng W, Fournier G, Mayhew GF, Plunkett G 3rd, Rose DJ, Darling A, et al. (2003) Complete genome sequence and comparative genomics of *Shigella flexneri* serotype 2a strain 2457T. Infect Immun. 71:2775-86.

80. Bolotin A, Wincker P, Mauger S, Jaillon O, Malarme K, Weissenbach J, Ehrlich SD, Sorokin A (2001) The complete genome sequence of the lactic acid bacterium *Lactococcus lactis* ssp. lactis IL1403. Genome Res. 11:731-53.

81. Ogata H, Audic S, Renesto-Audiffren P, Fournier PE, Barbe V, Samson D, Roux V, Cossart P, Weissenbach J, Claverie JM, et al. (2001) Mechanisms of evolution in *Rickettsia conorii* and *R. prowazekii*. Science 293:2093-8.

82. Nierman WC, Feldblyum TV, Laub MT, Paulsen IT, Nelson KE, Eisen JA, Heidelberg JF, Alley MR, Ohta N, Maddock JR, et al. (2001) Complete genome sequence of *Caulobacter crescentus*. Proc Natl Acad Sci U S A. 98:4136-41.

83. Kaneko T, Nakamura Y, Wolk CP, Kuritz T, Sasamoto S, Watanabe A, Iriguchi M, Ishikawa A, Kawashima K, Kimura, T, et al. (2001) Complete genomic sequence of the filamentous nitrogen-fixing *cyanobacterium Anabaena* sp. strain PCC 7120. DNA Res. 8:205-13; 227-53.

84. Nakamura Y, Kaneko T, Sato S, Ikeuchi M, Katoh H, Sasamoto S, Watanabe A, Iriguchi M, Kawashima K, Kimura T, Kishida Y, Kiyokawa C, Kohara M, Matsumoto M, Matsuno A, Nakazaki N, Shimpo S, Sugimoto M, Takeuchi C, Yamada M and Tabata S (2002) Complete genome structure of the thermophilic cyanobacterium *Thermosynechococcus elongatus* BP-1. DNA Res. 9 (4), 123-130.

85. Bao Q, Tian Y, Li W, Xu Z, Xuan Z, Hu S, Dong W, Yang J, Chen Y, Xue Y, et al. (2002) A complete sequence of the *T. tengcongensis* genome. Genome Res.12:689-700.

86. Gil R, Silva FJ, Zientz E, Delmotte F, Gonzalez-Candelas F, Latorre A, Rausell C, Kamerbeek J, Gadau J, Holldobler B, et al. (2003) The genome sequence of *Blochmannia floridanus*: comparative analysis of reduced genomes. Proc Natl Acad Sci U S A.100:9388-93.

87. Dufresne A, Salanoubat M, Partensky F, Artiguenave F, Axmann IM, Barbe V, Duprat S, Galperin MY, Koonin EV, Le Gall F, et al. (2003) Genome sequence of the cyanobacterium *Prochlorococcus marinus* SS120, a nearly minimal oxyphototrophic genome. Proc Natl Acad Sci U S A.100:10020-5.

88. Rocap G, Larimer FW, Lamerdin J, Malfatti S, Chain P, Ahlgren NA, Arellano A, Coleman M, Hauser L, Hess WR, et al. (2003) Genome divergence in two *Prochlorococcus* ecotypes reflects oceanic niche differentiation. Nature 424:1042-7.

89. Baar C, Eppinger M, Raddatz G, Simon J, Lanz C, Klimmek O, Nandakumar R, Gross R, Rosinus A, Keller H, et al. (2003) Complete genome sequence and analysis of *Wolinella succinogenes*. Proc Natl Acad Sci U S A.100:11690-5.

90. Duchaud E, Rusniok C, Frangeul L, Buchrieser C, Givaudan A, Taourit S, Bocs S, Boursaux-Eude C, Chandler M, Charles JF, et al. (2003) The genome sequence of the entomopathogenic bacterium *Photorhabdus luminescens*. Nat Biotechnol. 21:1307-13.

91. Makino K, Oshima K, Kurokawa K, Yokoyama K, Uda T, Tagomori K, Iijima Y, Najima M, Nakano M, Yamashita A, et al. (2003) Genome sequence of *Vibrio parahaemolyticus*: a pathogenic mechanism distinct from that of *V cholerae*. Lancet. 361:743-9.

92. Kim YR, Lee SE, Kim CM, Kim SY, Shin EK, Shin DH, Chung SS, Choy HE, Progulske-Fox A, Hillman JD, Handfield M and Rhee JH (2003) Characterization and Pathogenic Significance of *Vibrio vulnificus* Antigens Preferentially Expressed in Septicemic Patients. Infect. Immun. 71:5461-5471.
